# Molecular Typing and Variations in Amount of *tst* Gene Expression of TSST-1-Producing Clinical *Staphylococcus aureus* Isolates

**DOI:** 10.3389/fmicb.2019.01388

**Published:** 2019-06-19

**Authors:** Huanqiang Zhao, Su Xu, Han Yang, Chunyan He, Xiaogang Xu, Fupin Hu, Wen Shu, Fang Gong, Chuanling Zhang, Qingzhong Liu

**Affiliations:** ^1^Department of Clinical Laboratory, Shanghai General Hospital, Shanghai Jiaotong University School of Medicine, Shanghai, China; ^2^Institute of Antibiotics, Huashan Hospital, Fudan University, Shanghai, China; ^3^Department of Clinical Laboratory, the Third Hospital Affiliated to Nantong University, Wuxi, China; ^4^Department of Clinical Laboratory, Xiaoshan Hospital, Hangzhou, China

**Keywords:** virulence regulators, *Staphylococcus aureus*, toxic shock syndrome toxin-1, MRSA, gene expression, molecular typing

## Abstract

The toxic shock syndrome toxin-1 (TSST-1), encoded by *tst* gene, has been proposed to cause staphylococcal toxic shock syndrome (TSS) in a susceptible host, which highlights the need to evaluate the level of *tst* gene expression and molecular genetic characteristics of the *tst*-positive isolates. A total of 916 *S. aureus* isolates collected from seven hospitals in China were screened for the *tst* gene. The *tst* positive isolates were characterized by *spa*, SCC*mec*, PFGE, and *agr* typing. Representative strains were also subjected to MLST typing. qRT-PCR was used to quantify *tst* and major virulence regulator genes expression. We also sequenced the regions of promoter and open reading frame (ORF) of *tst* to investigate whether they correlate with the variation in *tst* expression. We found 208 (22.7%) of surveyed isolates including 198 (29.8%) of MRSA and 10 (4.0%) of MSSA isolates harbored the *tst* gene. The most common clone among *tst* positive MRSA isolates belonged to ST5 (CC5)-*agr*2-t002-SCC*mec*II. The amount of *tst* mRNA varied 8.4-folds among clinical *S. aureus* isolates. Sequencing the *tst* promoter revealed a base T deletion in *tst* high expressed isolates. As for major virulence regulators, *srrA, sarT, RNAIII*, and *ccpA* in four *tst* differentially expressed strains were detected to be highly expressed, respectively. Our study revealed high prevalence of ST5 (CC5)-*agr*2-t002-SCC*mec*II clone among *tst* positive MRSA in hospitals from China. The levels of *tst* expression among clinical *S. aureus* isolates varied, which may be associated with *tst* promoter and variations in specific virulence regulators.

## Introduction

*Staphylococcus aureus* (*S. aureus*), as the ubiquitous human pathogen, causes some of the most severe infections in both hospital and community settings. In China, *S. aureus* has become the main cause of infective osteomyelitis, endocarditis and sepsis (Liu et al., 2014; Yan et al., 2015; Xu et al., 2016). Relying on the selective expression of virulence factors that facilitate tissue invasion, adhesion or immune evasion, *S. aureus* can colonize almost any human tissue site and survive under highly variable conditions (Iwatsuki et al., 2006; Gorwitz et al., 2008).

*S. aureus* superantigens (SAgs), remarkably resistant to heat, acids, proteolysis and desiccation, are an extraordinary family of non-glycosylated low-molecular-weight exoproteins (Spaulding et al., 2013). The toxins of this family have the capacity to trigger excessive and non-conventional T-cell activation and cytokines release, and consequently interfere with immune system function systemically (Spaulding et al., 2013; Kulhankova et al., 2014). The biological toxicity of SAgs makes them be critical contributors causing life-threatening infections. The toxic shock syndrome toxin-1 (TSST-1), encoded by *tst* gene, is a significant member of SAgs and may lead to staphylococcal toxic shock syndrome (TSS) in a susceptible host (Spaulding et al., 2013).

It has been repeatedly documented that 30 to 40% of the population is asymptomatically colonized by *S. aureus* strains at one or more of their body sites (Blot et al., 1998; Spaulding et al., 2013), and approximate 20% of this organism is TSST-1 producers (Lindsay et al., 1998), indicating a large disease potential. However, the TSS, including both menstrual and non-menstrual categories, is fortunately rarely seen (1–3/100,000) (Brosnahan and Schlievert, 2011). The relatively high rate of *tst*-positive *S. aureus* isolates coupled with the low incidence of TSS strongly suggests that sufficient *tst* expression causes disease only under the appropriate environmental and/or genetic regulation control. Since the virulence of microbe may be dependent on the amount of toxins production (Bronner et al., 2004), quantifying the TSST-1 production by *S. aureus* strains contributes to offer a basis for developing the reasonable countermeasures to the *tst*-positive *S. aureus* infection control. It has been reported that the amounts of TSST-1 produced by clinical MRSA isolates differed up to 170-folds (Nagao et al., 2009). However, the specific regulatory factors or systems accounting for this diversifying gene expression among clinical *S. aureus* strains remain uncertain.

In this study, we aimed to (i) elucidate genetic background of the *tst*-positive *S. aureus* strains collected from seven hospitals in China, (ii) detect the difference at mRNA level of *tst in vitro*, and (iii) try to find the possible cause of the differential expression of *tst*.

## Materials and Methods

### Bacterial Strains

Two hundred and eight *tst*-positive *S. aureus* isolates (including 198 MRSA and 10 MSSA) were screened from 7 hospitals collection in Shanghai and Zhejiang province, China. This assemble of bacteria contained 916 non-duplicate *S. aureus* separated from clinical specimens during December 2008 and January 2013. Shanghai and Zhejiang are in the core position in the Yangtze River Delta, which is an economically advanced and densely populated area in China. Therefore, we think the isolates from these two regions may have a certain representativeness.

### DNA Extraction

Genomic DNAs of all the 916 *S. aureus* isolates were extracted with a TIANamp Bacterial DNA Kit (TIANGEN BIOTECH Co., Ltd., Beijing, China) according to the manufacturer's recommendations, with the modification of adding 10 μl of lysostaphin (1 mg/ml) and incubation at 37°C for 30 min for cell lysis. DNA amount and purity were tested using a NanoDrop spectrometer (Thermo Fisher Scientific, Waltham, MA, USA).

### Characterization of *tst*-Positive Isolates

All the 208 identified *tst*-positive *S. aureus* strains were characterized by staphylococcal protein A (*spa*) typing and pulsed-field gel electrophoresis (PFGE) typing as previously described (Mulvey et al., 2001; Koreen et al., 2004). In addition, the accessory gene regulator (*agr*) locus typing and staphylococcal cassette chromosome *mec* (SCC*mec*) typing had been previously carried out (Zhao et al., 2016) in all the *S. aureus* isolates with *tst* gene using methods described by Lina et al. (2003) and Zhang et al. (2005). Based on the diversity of origin and proportion of 30 to 45% of the isolates in each PFGE cluster, 76 (71 MRSA and 5 MSSA) representative isolates were chosen to be characterized by multi-locus sequence typing (MLST). The MLST was implemented by PCR amplification of internal fragments of seven housekeeping genes (*arcC, aroE, glpF, gmk, pta, tpi, and yqiL*) (Enright et al., 2000). MLST database (http://saureus.mlst.net/) was used to determine the allelic profile and the consequent sequence type (ST) of *tst*-positive isolates detected. The clustering of related STs into clonal complexes (CCs) was analyzed using eBURST (http://eburst.mlst.net/) as described previously (Feil et al., 2004).

### Total RNA Isolation and Quantitative Real-Time PCR (qRT-PCR)

Total RNA extraction and the subsequent qRT-PCR with specific primers were performed to analyze the expression of the *tst* gene and major virulence regulators in 32 isolates selected based on the proportion of 10 to 30% of the isolates in each PFGE cluster. For RNA extraction, overnight cultures were diluted 1:100 into 2 ml of TSB medium and grown to the post-exponential phase. Cells were deposited by the way of centrifugation. Total RNA samples were extracted using a MiniBEST Universal RNA Extraction Kit (TaKaRa) according to the manufacturer's instructions except a little change in the lysis step as described in the DNA extraction. The extracted RNA was quantified using a NanoDrop spectrometer (Thermo Fisher Scientific, Waltham, MA, USA). cDNA was synthesized with a PrimeScript® RT reagent Kit with gDNA Eraser (TaKaRa, Dalian, China) according to the manufacturer's protocol. The resulting cDNA products were stored at −20°C until use. The qRT-PCR was performed with 10 μl 2 × SYBR Premix Ex Taq (TaKaRa, Dalian, China), 0.4 μl ROX Reference Dye II (50 × ), 2 μl cDNA and 0.2 μM each of the forward and reverse primers (Table 1) in a final volume of 20 μl. The thermal cycling programs consisted of 30 s at 95°C, and then 40 cycles of 5 s at 95°C and 34 s at 60°C, followed by a dissociation step of 95°C for 15 s, 60°C for 1 min, and 95°C for 15 s on ABi 7500 Real Time PCR System (Applied Biosystems, Foster, CA, USA). The levels of expression of the target genes (*tst* and regulatory genes *RNAIII, sigB, sarA, ccpA, srrAB, rot*, and *sarT*) in the RNA samples were normalized on the basis of internal standards 16S rRNA. The specificity of the PCR was verified by melting curve analysis.

**Table 1:** qRT-PCR Primers used in this study.

**Primer name**	**Sequence**
RNAIII-F	TTCACTGTGTCGATAATCCA
RNAIII-R	TGATTTCAATGGCACAAGAT
*sarA*-F	ACATGGCAATTACAAAAATCAATGAT
*sarA*-R	TCTTTCTCTTTGTTTTCGCTGATG
*ccpA*-F	CCAAATGCTGTTGCTAGAGGTT
*ccpA*-R	TCTTCAAGTCCACGAGCAAGTT
*sigB*-F	TCTAAAGGACAATCACATCACGAAG
*sigB*-R	CCGTTCAAAGGACATATCGAATC
*srrA*-F	TAATGTTGCCTGAAATGGATGG
*srrA*-R	CAACACGGTTTGTTTCTTCACCT
*srrB*-F	AGCCGGCTAAATAGTGTCGT
*srrB*-R	ATGGCATTTTCGGTTTCTTG
*rot*-F	AACGACACTGTATTTGGGATTTTGC
*rot*-R	TTCGCTTTCAATCTCGCTGA
*sarT*-F	ATTTGAAAAGCAAGAGCAATATTAA
*sarT*-R	ATTTACCTTCATCATTTTTAAATACA
16s rRNA-F	TGAGATGTTGGGTTAAGTCCCGCA
16s rRNA-R	CGGTTTCGCTGCCCTTTGTATTGT
*tst*-F	TCGCTACAGATTTTACCCCTGT
*tst*-R	CGTTTGTAGATGCTTTTGCAGT

### Sequence Analysis of Variant *tst* Gene

*The tst*-gene DNA sequences from the 32 isolates selected with the principle mentioned above were sequenced and analyzed for the identification of mutations. The *tst* gene, including its promoter region, was amplified by PCR using specific primers (P-*tst*-F CTCAAAGATAGATTGACCAGCGATG, P-*tst*-R TTAATTTCTGCTTCTATAGTTT). All the products were sequenced in both directions by Shanghai Sangon Biotech. Sequences were aligned using CLUSTA L X 2.0.

### Statistical Analysis

All data were expressed as mean ± standard deviation (SD). Statistically significant differences were determined by ANOVA alone or with a LSD *post-hoc* test by SAS 9.3 for Windows software (SAS Institute Inc., NC, USA). In each case, statistical significance was indicated by *p* < 0.05.

## Results

### Characterization of *S. aureus* Harboring *tst* Gene

By sequence analysis, a total of 15 *spa* types were yielded among the 208 *tst*-positive strains, of which 8 *spa* types were related exclusively to MRSA, 2 exclusively to MSSA, and 5 to both. The most prevalent *spa* type in MRSA was t002 (82.3%, 163/198), followed by t2460 (4.5%, 9/198) and t311 (3.5%, 7/198). Other *spa* types identified in MRSA were detailed as follows: t010 (4/198, 2.0%), t548 (3/198, 1.5%), t037 (2/198, 1.0%), t034 (2/198, 1.0%), t148 (2/198, 1.0%), t242 (2/198, 1.0%), t318 (1/198, 0.5%), t570 (1/198, 0.5%), t796 (1/198, 0.5%), t1751 (1/198, 0.5%). Most of *tst*-positive MSSA strains were also t002, accounting for 40% (4/10), and the 6 remaining isolates were t034, t091, t148, t2461, t548, t570, respectively.

**Figure 1:**
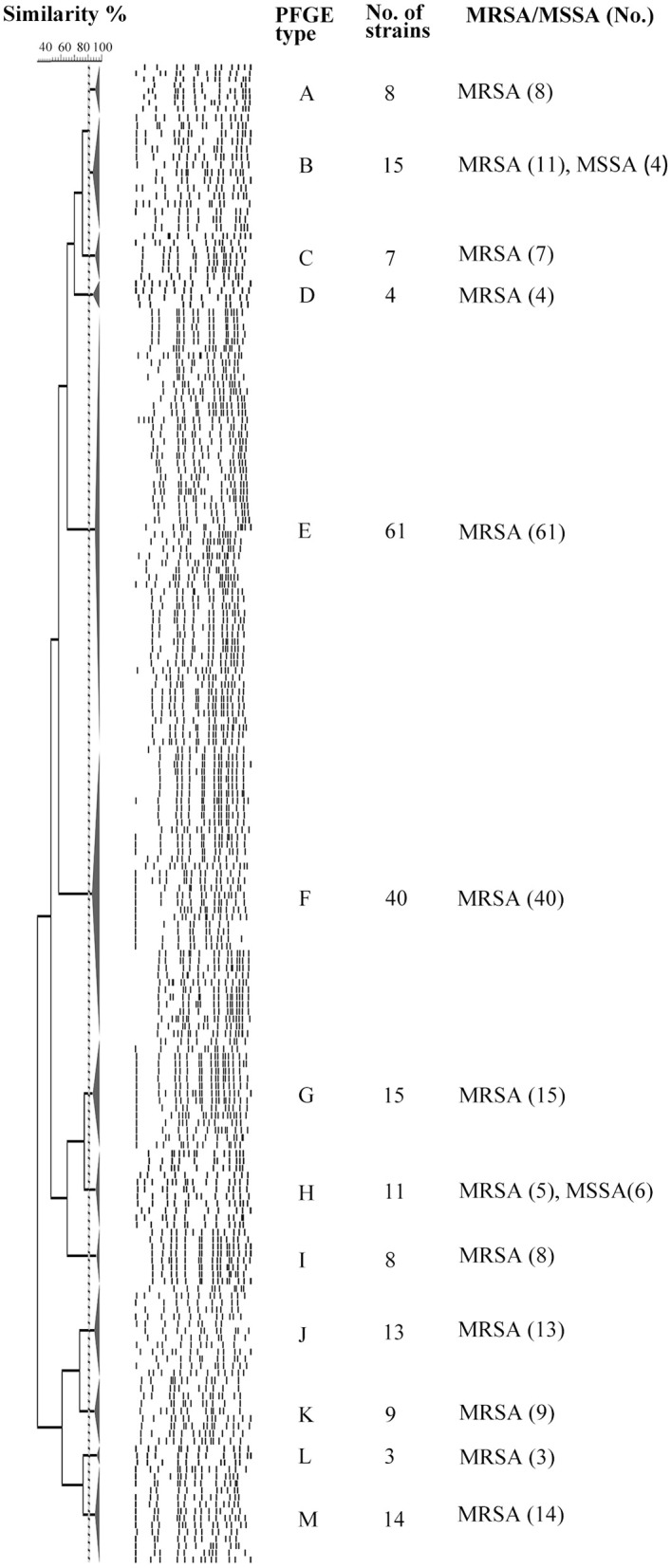
PFGE analysis of 208 *tst* gene positive *S. aureus* isolates digested with SmaI. This dendrogram shows the 208 *S. aureus* strains were clustered into 13 genomic groups (A-M).

The 208 *tst*-positive isolates were classified into 13 PFGE types that were designated by symbols A to M (Figure 1). Most of isolates were clustered into PFGE types E and F, accounting for 29.3% (61/208) and 19.2% (40/208), respectively. Of note, all the MSSA carrying *tst* gene from different hospitals were grouped into the clusters B and H (Figure 1). Therefore, the high genetic homology of these isolates may demonstrate the existence of a prevalent *tst*-positive MSSA strain.

The 76 (71 MRSA and 5 MSSA) representative isolates selected on the basis of the aforementioned PFGE dendrogram were detected to contain 9 different STs, of which ST5 (78.9%, 60/76) was most frequently identified, followed by ST2590 (9.2%, 7/76), ST7 (2.6%, 2/76), ST72 (2.6%, 2/76), and 5 additional STs, namely ST59, ST764, ST1860, ST188 and ST239 (1.3%, 1/76 each) (Table 2). Notably, except that 4 MRSA belonging to ST7 (2.6%, 2/76), ST59 (1.3%, 1/76), and ST239 (1.3%, 1/76) clones were clustered to CC7, CC59 and CC8, respectively, all the remaining isolates were clustered to CC5 (94.7%, 72/76). These results indicated a high homogeneity among the *tst*-positive isolates with regard to their MLST types.

**Table 2:** Characteristics of 208 *tst* gene positive *S. aureus* isolates.

**Isolate ID**	**Location**	**SCC*mec*-*agr* (Zhao et al., 2016)**	***spa***	**PFGE cluster**	**ST**	**CC**	**Isolate ID**	**Location**	**SCC*mec*-*agr* (Zhao et al., 2016)**	***spa***	**PFGE cluster**	**ST**	**CC**
PT56	Shanghai	II-2	t002	A			PT42	Shanghai	N-2	t002	F		
PT64	Shanghai	II-2	t002	A			PT88	Shanghai	II-2	t002	F	ST5	CC5
PT128	Shanghai	II-2	t002	A			PT89	Shanghai	II-2	t002	F	ST5	CC5
PT130	Shanghai	II-2	t002	A			PT113	Shanghai	II-2	t034	F		
PT140	Shanghai	N-1	t796	A	ST7	CC7	PT120	Shanghai	II-2	t002	F		
PT157	Shanghai	II-2	t034	A			PT121	Shanghai	II-2	t002	F		
PT528	Shanghai	II-2	t002	A	ST5	CC5	PT138	Shanghai	II-2	t002	F		
LS2164	Zhejiang	II-2	t311	A	ST5	CC5	PT156	Shanghai	II-2	t002	F		
PT3	Shanghai	II-2	t002	B			PT189	Shanghai	II-2	t002	F		
PT4	Shanghai	II-N	t002	B			PT192	Shanghai	II-2	t002	F		
PT94	Shanghai	II-2	t002	B			PT220	Shanghai	II-2	t002	F		
PT98	Shanghai	II-2	t002	B			PT228	Shanghai	II-2	t002	F	ST5	CC5
PT114	Shanghai	II-2	t002	B			PT233	Shanghai	II-2	t2460	F		
PT202	Shanghai	II-2	t002	B			PT249	Shanghai	II-2	t091	F		
PT238	Shanghai	II-2	t002	B	ST5	CC5	PT262	Shanghai	II-2	t002	F		
PT250	Shanghai	II-2	t548	B			PT287	Shanghai	II-2	t002	F		
PT306	Shanghai	II-2	t002	B			PT294	Shanghai	II-2	t002	F		
PT333	Shanghai	II-2	t2460	B			PT317	Shanghai	II-2	t002	F		
LS1200	Zhejiang	II-2	t311	B	ST5	CC5	PT368	Shanghai	II-2	t002	F		
PTs3	Shanghai	/-2	t002	B			PT381	Shanghai	II-2	t002	F		
PTs87	Shanghai	/-1	t002	B	ST188	CC5	PT469	Shanghai	II-2	t002	F		
LS1956	Zhejiang	/-1	t2461	B	ST72	CC5	PT474	Shanghai	II-2	t002	F		
SP116	Shanghai	/-2	t570	B	ST5	CC5	PT545	Shanghai	II-2	t002	F	ST5	CC5
PT369	Shanghai	II-1	t002	C	ST5	CC5	PT565	Shanghai	II-2	t002	F	ST5	CC5
PT382	Shanghai	II-2	t002	C			PT567	Shanghai	II-2	t010	F	ST5	CC5
PT443	Shanghai	II-2	t002	C			HK1011	Shanghai	II-2	t002	F	ST5	CC5
HK471	Shanghai	II-2	t002	C	ST2590	CC5	PT572	Shanghai	II-2	t242	F		
FP486	Shanghai	II-2	t002	C	ST2590	CC5	LS1927	Zhejiang	II-2	t002	F	ST5	CC5
FP547	Shanghai	II-2	t002	C			LS2028	Zhejiang	II-2	t311	F	ST5	CC5
XS12	Zhejiang	II-2	t318	C			FP490	Shanghai	II-2	t002	F	ST5	CC5
PT1	Shanghai	II-N	t002	D			FP540	Shanghai	II-2	t002	F	ST5	CC5
PT124	Shanghai	II-2	t002	D	ST2590	CC5	PT13	Shanghai	II-2	t002	G	ST5	CC5
PT194	Shanghai	II-2	t002	D	ST2590	CC5	PT25	Shanghai	II-2	t148	G	ST5	CC5
PT462	Shanghai	II-2	t2460	D	ST5	CC5	PT63	Shanghai	II-2	t002	G		
PT26	Shanghai	N-2	t002	E	ST5	CC5	PT79	Shanghai	II-2	t002	G		
PT27	Shanghai	II-2	t002	E	ST5	CC5	PT82	Shanghai	II-2	t002	G	ST5	CC5
PT41	Shanghai	III-2	t002	E	ST5	CC5	PT86	Shanghai	II-2	t002	G	ST5	CC5
PT44	Shanghai	II-2	t002	E	ST5	CC5	PT111	Shanghai	II-2	t002	G		
PT71	Shanghai	II-2	t002	E	ST5	CC5	PT143	Shanghai	II-2	t002	G		
PT72	Shanghai	II-2	t002	E	ST5	CC5	PT193	Shanghai	II-2	t002	G		
PT73	Shanghai	II-2	t002	E	ST5	CC5	PT209	Shanghai	II-2	t2460	G	ST5	CC5
PT80	Shanghai	II-2	t002	E	ST5	CC5	PT258	Shanghai	II-2	t002	G		
PT91	Shanghai	II-2	t002	E	ST5	CC5	PT365	Shanghai	II-2	t002	G		
PT92	Shanghai	II-2	t002	E			PT374	Shanghai	II-2	t002	G		
PT96	Shanghai	II-2	t002	E			FP500	Shanghai	II-2	t002	G		
PT99	Shanghai	II-2	t002	E			FP541	Shanghai	II-2	t242	G		
PT101	Shanghai	II-2	t002	E			PT107	Shanghai	N-2	t002	H	ST2590	CC5
PT102	Shanghai	II-2	t548	E			PT127	Shanghai	II-2	t002	H		
PT104	Shanghai	II-2	t002	E			PT148	Shanghai	II-2	t002	H		
PT105	Shanghai	II-2	t002	E			PT175	Shanghai	II-2	t570	H		
PT106	Shanghai	II-2	t002	E			PT180	Shanghai	N-2	t002	H	ST2590	CC5
PT112	Shanghai	II-2	t002	E			TR5865	Shanghai	/-2	t002	H	ST5	CC5
PT117	Shanghai	II-2	t002	E			FP1726	Shanghai	/-2	t548	H		
PT118	Shanghai	II-2	t002	E			SJ0951	Shanghai	/-1	t148	H	ST72	CC5
PT122	Shanghai	II-2	t002	E			FP131007	Shanghai	/-1	t091	H		
PT123	Shanghai	II-2	t002	E			PTs2	Shanghai	/-2	t002	H		
PT133	Shanghai	II-2	t002	E			LS244	Zhejiang	/-1	t034	H		
PT137	Shanghai	II-2	t002	E			PT39	Shanghai	II-2	t002	I	ST764	CC5
PT139	Shanghai	II-2	t002	E			PT59	Shanghai	II-2	t002	I		
PT145	Shanghai	II-2	t002	E			PT67	Shanghai	II-2	t002	I		
PT153	Shanghai	II-2	t002	E			PT68	Shanghai	II-2	t002	I	ST5	CC5
PT154	Shanghai	II-2	t002	E			PT75	Shanghai	II-2	t002	I		
PT169	Shanghai	II-2	t002	E			PT109	Shanghai	II-2	t002	I		
PT198	Shanghai	II-2	t002	E	ST5	CC5	PT142	Shanghai	II-2	t002	I	ST5	CC5
PT201	Shanghai	III-2	t002	E	ST239	CC8	PT197	Shanghai	II-2	t002	I		
PT211	Shanghai	II-2	t010	E			PT16	Shanghai	II-2	t002	J		
PT212	Shanghai	II-2	t002	E			PT18	Shanghai	II-2	t002	J		
PT214	Shanghai	II-2	t002	E	ST5	CC5	PT87	Shanghai	II-2	t002	J		
PT217	Shanghai	II-2	t002	E	ST5	CC5	PT187	Shanghai	II-2	t002	J		
PT224	Shanghai	II-2	t002	E			PT232	Shanghai	II-2	t002	J	ST5	CC5
PT235	Shanghai	II-2	t002	E	ST5	CC5	PT251	Shanghai	II-2	t002	J		
PT244	Shanghai	II-2	t002	E	ST7	CC7	PT255	Shanghai	II-2	t002	J		
PT247	Shanghai	II-2	t002	E			PT300	Shanghai	II-2	t002	J		
PT259	Shanghai	II-2	t002	E			PT388	Shanghai	II-2	t002	J		
PT264	Shanghai	II-2	t002	E			PT395	Shanghai	II-2	t002	J		
PT271	Shanghai	II-2	t002	E			LS44	Zhejiang	II-2	t311	J	ST5	CC5
PT272	Shanghai	II-2	t002	E			LS684	Zhejiang	II-2	t311	J	ST5	CC5
PT283	Shanghai	II-2	t002	E			LS1775	Zhejiang	II-2	t311	J	ST5	CC5
PT286	Shanghai	II-2	t002	E			PT48	Shanghai	II-2	t548	K		
PT291	Shanghai	II-2	t002	E			PT76	Shanghai	II-2	t002	K		
PT308	Shanghai	II-2	t2460	E			PT81	Shanghai	II-2	t002	K		
PT311	Shanghai	II-2	t002	E	ST5	CC5	PT240	Shanghai	II-2	t002	K	ST5	CC5
PT325	Shanghai	II-2	t002	E			PT289	Shanghai	II-2	t002	K		
PT349	Shanghai	II-N	t002	E			PT440	Shanghai	II-2	t2460	K		
PT361	Shanghai	III-2	t002	E			PT489	Shanghai	II-2	t002	K		
PT372	Shanghai	N-2	t010	E			PT587	Shanghai	II-2	t002	K	ST1860	CC5
PT377	Shanghai	II-2	t002	E			XP114	Shanghai	II-2	t002	K	ST5	CC5
PT389	Shanghai	II-2	t002	E			RJ24	Shanghai	II-2	t002	L	ST5	CC5
PT393	Shanghai	II-2	t002	E			PT61	Shanghai	II-2	t002	L		
PT439	Shanghai	II-2	t002	E			FP560	Shanghai	N-2	t002	L	ST5	CC5
PT541	Shanghai	II-2	t002	E	ST5	CC5	PT23	Shanghai	II-2	t002	M	ST5	CC5
PT548	Shanghai	II-2	t2460	E	ST5	CC5	PT30	Shanghai	II-2	t002	M		
XP37	Shanghai	II-2	t010	E	ST5	CC5	PT78	Shanghai	II-2	t002	M		
LS373	Zhejiang	IVa-1	t1751	E	ST59	CC59	PT234	Shanghai	II-2	t002	M	ST5	CC5
LS1887	Zhejiang	II-2	t311	E	ST5	CC5	PT375	Shanghai	II-2	t002	M		
PT2	Shanghai	II-2	t002	F			PT391	Shanghai	II-2	t002	M		
PT8	Shanghai	II-2	t002	F	ST5	CC5	PT400	Shanghai	II-2	t002	M		
PT10	Shanghai	II-2	t002	F			PT448	Shanghai	II-2	t002	M		
PT12	Shanghai	II-2	t002	F	ST2590	CC5	PT464	Shanghai	II-2	t2460	M		
PT15	Shanghai	II-2	t148	F			PT525	Shanghai	II-2	t002	M	ST5	CC5
PT32	Shanghai	II-2	t091	F			PT554	Shanghai	II-2	t2460	M		
PT33	Shanghai	II-2	t002	F			PT561	Shanghai	II-2	t002	M	ST5	CC5
PT34	Shanghai	II-2	t002	F	ST5	CC5	PT676	Shanghai	II-2	t002	M	ST5	CC5
PT36	Shanghai	II-2	t002	F	ST5	CC5	FP473	Shanghai	II-2	t002	M		

*N, nontypeable; /, not applicable; PFGE, pulsed-feld gelelectrophoresis; SCCmec, staphyloccoccal cassette chromosome mec element; ST, sequence type*.

As is apparent from Table 2, all ST2590 strains expressed *agr* type II and *spa* type 002. Among the 60 strains of ST5, 59 (98.3%, 59/60) were detected as *agr* type II, while the one remaining strain belonged to *agr* type I. Except two untypeable and one SCC*mec* III isolates, all the ST5 MRSA strains harbored SCC*mec* II. Both the *agr* and SCC*mec* types of all the *spa* t311 isolates were detected as type II, while their MLST types were identified as ST5. Five SCC*mec*-untypeable MRSA strains belonged to CC5-*spa* t002-*agr* II (4 isolates) and CC7-*spa* t796-*agr* I (1 isolate).

We can summarize from Table 2 that the most common clone among MRSA isolates belonged to ST5 (CC5)-*agr* II-*spa* t002-SCC*mec* II (53.9%, 41/76). While the predominant genotype of MSSA was not found because of the less *S. aureus* isolates.

### Direct Transcript Analysis of *tst* Gene

**Figure 2:**
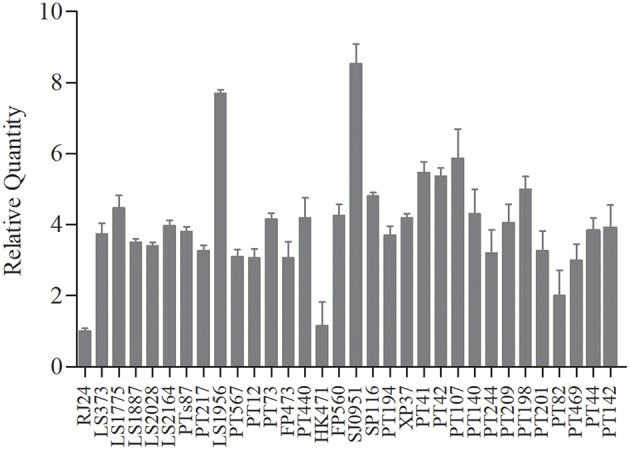
The transcriptional levels of *tst* gene in 32 chosen *S. aureus* strains representative. No obvious variations in the expression levels of *tst* gene among most of the strains, except two MRSA strains (L-RJ24 and L-HK471) with higher and two MSSA strains (H-LS1956 and H-SJ0951) with lower *tst* mRNA abundances. Data are expressed as the means ± standard deviation (SD). The experiment was independently repeated three times.

We performed qRT-PCR with *tst*-specific primers to assess the *ts*t/l6S ratio on the post-exponential bacterial pellets (32 representative *S. aureus* isolates). The results showed no obvious variations in the expression levels of *tst* gene among most of the chosen strains, except several with a significantly different *tst* mRNA level (Figure 2). The amount of *tst* mRNA varied 8.4-folds among clinical MRSA isolates in the present study. Two strains with highest and lowest *tst* mRNA abundances each were chosen to probe into the cause of the differential expression. Interestingly, the two strains (L-RJ24 and L-HK471) lowly expressing *tst* gene belonged to the same CC, *spa*, SCC*mec* and *agr* types (CC5-*agr*2-t002-SCC*mec*II), and the two isolates (H-LS1956 and H-SJ0951) with high amount of *tst* mRNA were identified as MSSA.

### Expression of Major Virulence Regulators

**Figure 3:**
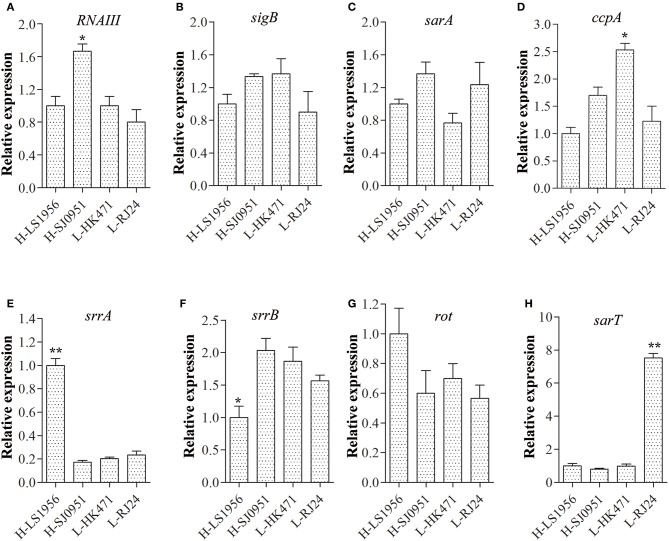
Expression of major virulence regulator genes including *RNAIII* **(A)**, *sigB* **(B)**, *sarA* **(C)**, *scpA* **(D)**, *srrA* **(E)**, *srrB* **(F)**, *rot* **(G)**, and *sar*T **(H)** in four *tst* differentially expressed *S. aureus* isolates. *RNAIII* in strain H-SJ0951, *ccpA* in strain L-HK471, *srrA* in strain H-LS1956, and *sarT* in strain L-RJ24 were detected to be highly expressed and *srrB* in strain H-LS1956 were detected to be lowly expressed in the post-exponential growth phase. Data are expressed as the means ± standard deviation (SD). Statistically significant differences were determined by ANOVA with LSD *post-hoc* test. *p < 0.05, ** *p* < 0.01 compared with the closest *tst* expression of one of the three strains. The experiment was independently repeated three times.

Since RNAIII, SigB, SarA, CcpA, SrrAB, Rot, and SarT have been previously shown to have effects on *tst* transcription (Schmidt et al., 2001; Pragman et al., 2007; Seidl et al., 2008; Andrey et al., 2010, 2015), we measured the transcript levels of these modulator genes in four differentially expressed isolates by qRT-PCR (Figures 3A–H). The results showed comparable expression levels of *sigB, sarA*, and *rot* in the four strains (Figures 3B,C,G). Although both strains H-LS1956 and H-SJ0951 produced large amounts of *tst* mRNA, the expressions of the major virulence regulator genes did not consistently vary in any way from that of strains L-RJ24 and L-HK471, and vice versa. However, *RNAIII* in strain H-SJ0951 (Figure 3A), *ccpA* in strain L-HK471 (Figure 3D), *srrA* in strain H-LS1956 (Figure 3E) and *sarT* in strain L-RJ24 (Figure 3H) were detected to be highly expressed, and *srrB* in strain H-LS1956 (Figure 3F) was detected to be lowly expressed in the post-exponential growth phase.

### Detection of Mutations in Open Reading Frame (ORF) and Promoter of *tst* Gene

**Figure 4:**
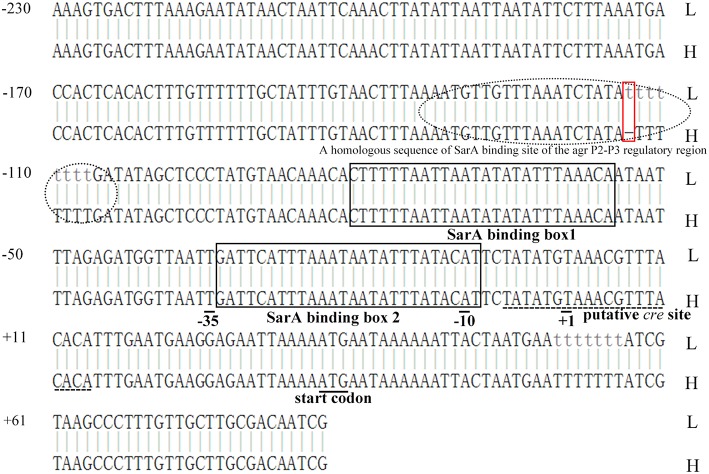
Alignment of *tst* promoters in *tst* gene high- and low-expression isolates. H, *tst* high-expression strains; L, *tst* low-expression strains. Two isolates (H-LS1956 and H-SJ0951) with the *tst* gene high expression have a base T deletion in the promoter region (nt-114, red rectangular box). This region is AT-rich and the homologous sequence of the *agr* P2-P3 regulatory site SarA binding (dotted oval) (Chan and Foster, 1998b), and is located in the upstream of SarA binding box 1/2 (black rectangular boxes) and a putative catabolite-responsive element (*cre*) region (dashed line) (Seidl et al., 2008).

To determine how the promoter of *tst* gene and the structure of TSST-1 itself affect the amount of *tst* mRNA, the region containing the ORF and promoter of *tst* gene in the four above-mentioned isolates were sequenced. A comparison of the ORF nucleotide sequences from the four strains with the corresponding sequence of the *tst* gene from *S. aureus* strain N315 (GenBank accession no. BA000018.3) revealed no changes. While one mutation for *tst* promoter was detected between the high and low *tst* expressed isolates (GenBank accession no. MK537300 and MK537301) (Figure 4). More specifically, a base T deleted in the promoter region (nt-114) was detected in the two isolates with high-expressed *tst* gene. The mutation occurred in an AT-rich region, which is the homologous sequence of the *agr* P2-P3 regulatory site SarA binding (Chan and Foster, 1998b) (Figure 4). And it is located in the upstream of SarA binding box 1/2 and a putative catabolite-responsive element (*cre*) region (Seidl et al., 2008).

## Discussion

TSST-1 is a major virulence factor in *S. aureus* infections. Here, 208 *tst*-positive *S. aureus* clinical isolates collected from multiple hospitals in China were characterized. We found ST5 (CC5)-*agr* II-t002-SCC*mec*II was the most common clone among MRSA. Moreover, our results indicated promoter mutation may be one of the factors resulting in the *tst* gene differential expression. However, it is still need to be verified by site-directed mutagenesis studies. The epidemiology of *tst*-positive MRSA showed regional variations worldwide. A cross-sectional study in Shiraz, Iran has found 18.1% of surveyed MSSA and 11.6% of MRSA possessed *tst* gene (Motamedifar et al., 2015). Another study from Japan reported that up to 75% of MRSA harbored *tst* gene, 96.7% of which belonged to *agr* 2 (Nagao et al., 2009). In China, the regional differences also exist. A multicenter study showed 31.4% of *S. aureus* surveyed were *tst* positive, and CC398, CC15, and CC188 were the most common (He et al., 2013). The CC5 clone is not usually observed in *tst* gene positive *S. aureus* isolates in China but reports of this clone are now emerging. A recent study from Suzhou, a city near Shanghai, reported that *tst* was detected in 18.0% of 150 isolates collected, and is mostly associated with CC5-t002 (Wang et al., 2017). In our strain collection, 29.8% (198/665) of MRSA and 4.0% (10/251) of MSSA isolates were *tst* gene positive, and ST5 (CC5) isolates (Table 2) with the genotype consisting of *agr* type 2, *spa* type t002, SCC*mec* type II (Zhao et al., 2016), accounted for 53.9 % of 76 representative isolates chosen to be characterized by MLST. It appears therefore that *tst* prevalence rate is regionally different and CC5 may represent a newly emerging clone in east of China. Additionally, all the chosen *tst* positive MSSA isolates were CC5 clone, indicating the CC5 *tst* carrying MRSA likely evolved from the similar CC5 *tst* carrying MSSA clone.

Due to the high percentage of *S. aureus* isolates carrying *tst* gene, understanding of *tst* expression and the possible expression regulatory mechanism appears to be important. Nagao et al. (2009) identified 170-fold variation in the amount of TSST-1 produced among clinical MRSA isolates. While our results revealed that *tst* expression varied 8.4-folds on transcriptional level among our clinical *S. aureus* isolates representative. The magnitude of this difference may derive from detection methods (qRT-PCR vs. western blot). Additionally, we found strains with high *tst* expression level only accounted for a small part of the isolates detected, generally explaining the phenomenon that relatively high rate of *S. aureus* harboring *tst* gene with the low incidence of TSS. This also supported the notion that TSST-1 expression is under the rigorous genetic regulation (Bronner et al., 2004).

MSSA strains have greater potential to secrete toxins, such as Panton-Valentine leukocidin (PVL), than MRSA (Varshney et al., 2010). The cause may be that MSSA isolates have less genetic fitness burden due to the lack of the SCC*mec* element carriage. However, conflicting conclusions exist in the balance of TSST-1 production and the SCC*mec* element carriage in *S. aureus*. Schmitz et al. (1997) have investigated the toxin production of clinical *S. aureus* isolates and found the TSST-1 expression was independent of the sensitivity of *S. aureus* to methicillin. While a recent study showed that the *tst*-positive CC30 MSSA strains produced more TSST-1 toxin when compared with *tst*-positive CC30 MRSA isolates (Sharma et al., 2018), which is consistent with the findings of this study: the two MSSA isolates (H-LS1956 and H-SJ0951) expressed obviously higher mRNA levels of *tst* than MRSA with the same genetic background. The contradiction may arise from whether to uniform genetic background of *S. aureus* when comparing TSST-1 production and carriage of SCC*mec* element.

Various environmental factors including glucose, oxygen, magnesium ions, α and β chains of hemoglobin, a range of antibiotics (e. x. nafcillin and clindamycin) and TSST-1 itself have been proved to influence the expression of TSST-1 (Kass, 1989; Chan and Foster, 1998a; Yarwood and Schlievert, 2000; Pragman et al., 2007; Schlievert et al., 2007; Stevens et al., 2007; Seidl et al., 2008). These environmental triggers affect TSST-1 expression via a large number of virulence regulators forming a complicated network in *S. aureus* (Andrey et al., 2015). The accessory gene regulator (*agr*) system and staphylococcal accessory regulator A (SarA) are thought to be prominent factors to modulate TSST-1 production. The *agr* system plays a central role in the pathogenesis of *S. aureus*, and its effector termed RNAIII has been shown to upregulate TSST-1 production (Recsei et al., 1986). SarA affects *tst* expression through binding to a certain element in *tst* gene promoter (Andrey et al., 2010). Other regulators controlling *tst* expression include sigma factor B (SigB) (Andrey et al., 2015), Staphylococcal respiratory response AB (SrrAB) (Pragman et al., 2004), Carbon catabolite protein A (CcpA) (Seidl et al., 2008), SarT (Schmidt et al., 2001), and repressor of toxin (Rot) (Andrey et al., 2015). However, these regulators are only studied in several type strains, whether they take effects in clinical isolates remains unclear. In the present study, we analyzed the expression of *RNAIII, sigB, sarA, ccpA, srrAB, rot*, and *sarT* in four *tst* differentially expressed strains, and found *srrA, srrB, RNAIII, sarT*, and *ccpA* were expressed unnormally in specific strains, indicating the regulatory networks in different strains may be not uniformly the same. In this study, we also sequenced the promoter and ORF region of *tst*. Consequently, a base T deletion located on an AT-rich region binding SarA in *tst* overexpression isolates was revealed, which was thought to be more likely to lead to *tst* high expression. Conversely, a previous study reported that no change was found in the sequences of promoter region among differentially expressed strains (Nagao et al., 2009). Overall, the previous and present results indicate that TSST-1 production is controlled in various and complex regulatory systems.

This study had several limitations. Firstly, this study lacks detailed clinical information about the patients, thus we cannot evaluate the association between outcome of patients and *tst* expression level. Secondly, we only found a mutation in *tst* promoter in high-expression isolates. Further study, including using dual luciferase report system to determine the function of mutation in *tst* promoter, will be needed. Thirdly, four differentially expressed virulence regulators in the four chosen isolates were revealed. Whereas, the mechanisms are still unclear and remain to be further demonstrated.

## Conclusions

In summary, although we observed a heterogeneity of *tst*-positive MRSA clones, type ST5 (CC5)-*agr*II-t002-SCC*mec*II was proved to be represented one dominated clone in the regions investigated. This highlights the need for prevention and control measures to prevent the spread of this type of strains in China, particularly in Shanghai and Zhejiang regions. Although the *tst* high-expressing strains rarely occur among the clinical *S. aureus* isolates, targeting TSST-1, such as using some protein synthesis inhibitory antibiotics or herbal extracts inhibiting the production of this toxin (Qiu et al., 2011, 2012; Hodille et al., 2017; Katahira et al., 2019), for the treatment of *S. aureus* infection can be considered as an alternative strategy because TSS caused by TSST-1 is fatal. Additionally, the expression of *tst* has a potential association with the mutation of its promoter and variations in specific virulence regulators expression. Therefore, sequence of the *tst* promoter and quantification of major virulence regulators expression may provide significant information in survey of MRSA infection.

## Author Contributions

QL designed and conceived the investigation. HZ, HY, and SX carried out the experiments. HZ, CH, XX, FH, WS, FG, and CZ analyzed the experiment data. HZ, SX, and QL wrote this manuscript. QL and HZ revised the article and approved the final version to be published.

## Key Contribution

These findings implied that ST5 (CC5)-*agr*2-t002-SCC*mec*II is the most prevalent *tst* positive *S. aureus* clone in the region investigated. In addition, data also showed that *tst* expression of clinical *S. aureus* isolates may be associated with *tst* promoter and variations in specific virulence regulators.

### Conflict of Interest Statement

The authors declare that the research was conducted in the absence of any commercial or financial relationships that could be construed as a potential conflict of interest.
